# Socioeconomic status and health disparities drive differences in accelerometer-derived physical activity in fatty liver disease and significant fibrosis

**DOI:** 10.1371/journal.pone.0301774

**Published:** 2024-05-09

**Authors:** Lucia Tabacu, Sajag Swami, Mark Ledbetter, Mohamad S. Siddiqui, Ekaterina Smirnova

**Affiliations:** 1 Dept. of Mathematics and Statistics, Norfolk, Virginia, United States of America; 2 Dept. of Mechanical and Industrial Engineering, Indian Institute of Technology Roorkee, Roorkee, India; 3 BWX Technologies, Nuclear Operations Group, Lynchburg, Virginia, United States of America; 4 Div. of Gastroenterology, Hepatology and Nutrition, Dept. of Internal Medicine, Virginia Commonwealth University School of Medicine, Richmond, Virginia, United States of America; 5 Dept. of Biostatistics, Virginia Commonwealth University School of Medicine, Richmond, Virginia, United States of America; Universidad Andres Bello, CHILE

## Abstract

**Background and aims:**

The cornerstone of clinical management of patients with nonalcoholic fatty liver disease (NAFLD) are lifestyle changes such as increasing physical activity (PA) aimed at improving cardiometabolic risk. To inform NAFLD prevention and treatment guidelines we aimed to: (i) quantify the role of PA on lowering the risk for NAFLD and fibrosis; (ii) characterize NAFLD and fibrosis association with PA in the context of socioeconomic environment.

**Methods:**

A sample of 2648 participants from the NHANES 2003–2006 was selected to develop survey weighted multivariable logistic regression models for predicting NAFLD and significant fibrosis, diagnosed non-invasively via fatty liver index (FLI) and fibrosis-4 (FIB-4) index. The PA measures were obtained from a hip-worn accelerometer.

**Results:**

The predictive model for NAFLD showed AUC of 0.687 and a decrease of 43% in NAFLD risk with moderate vigorous PA (MVPA) (OR = 0.569, p < 0.001). The predictive model for fibrosis had AUC of 0.755 and there was a 48% and a 70% decrease in significant fibrosis risk with MVPA (OR = 0.518, p = 0.022) and total log activity count (TLAC) (OR = 0.296, p = 0.017), respectively. Participants with NAFLD and NAFLD with fibrosis engage in declining PA. Despite having jobs with higher level of PA and participating in more moderate-to-vigorous PA, a larger proportion of Hispanics participants had NAFLD and significant fibrosis.

**Conclusions:**

These findings demonstrate the role of PA as a protective factor against the presence of NAFLD and significant fibrosis. Protective levels of PA in NAFLD differ by races.

## Introduction

Nonalcoholic fatty liver disease (NAFLD) is the most common cause of chronic liver disease in the United States, affecting nearly 1 in 3 Americans [1–3]. NAFLD is commonly associated with metabolic comorbid conditions such as diabetes, obesity, and cardiovascular disease [2, 4]. NAFLD can be classified further based on liver biopsy into either nonalcoholic fatty liver (NAFL) or nonalcoholic steatohepatitis (NASH) [5]. Patients with NAFL are at relatively low risk of fibrosis progression and cirrhosis, whereas patients with NASH are at considerably higher risk for fibrosis progression and cirrhosis.

As NAFLD is considered the hepatic manifestation of metabolic syndrome, its prevalence is considerably higher in patients with coexisting metabolic comorbidities such as obesity [1, 6]. NAFLD development is closely related to sedentary lifestyle and Western diet [7]. Given this association between obesity and NAFLD, multiple studies have targeted lifestyle modification aimed at increasing physical activity to treat NAFLD [8, 9]. Several studies have now demonstrated that structured physical activity improves hepatic fat content, irrespective of weight loss. Even modest amount of physical activity, compared to inactivity, is associated with reduced risk of NAFLD [10] and sedentary behavior is associated with higher risk of metabolic comorbidities including NAFLD [11–13]. Furthermore, previous studies connected lower socioeconomic status to higher rates of NAFLD and its severity [14] and found significant interactions between physical activity and socioeconomic status on NAFLD prevalence in a large NHANES III and NHANES 1999–2014 cohorts [15].

However, thus far, the literature evaluating the role of physical activity in diverse socioeconomic groups on NAFLD concentrated on self-reported physical activity, which can be affected by cognitive impairment associated with disease, age, and psychosocial factors [16–19]. While some previous literature examined the associations between accelerometer-measured physical activity and NAFLD [20] in NHANES 2003–2006 and further linked it to mortality outcomes [21–24], no study has yet connected objectively measured physical activity to NAFLD and further to fibrosis. Additionally, there is a lack of data regarding the association between the physical activity and fibrosis in diverse socioeconomic groups. To address these significant gaps in the literature, we sought to examine the relationship between the socioeconomic status in NHANES 2003–2006 cohort, which includes detailed information regarding physical activity measured objectively by accelerometers, socioeconomic status, and chronic liver disease. We hypothesized that presence and severity of NAFLD will correlate inversely with physical activity. Furthermore, this association will be more pronounced in individuals of lower socioeconomic status.

## Methods

### Study cohort

The NHANES is the ongoing study conducted by the Centers for Disease Control and Prevention (CDC) to monitor health of the US population [25]. The data collected includes demographic, socioeconomic, dietary, health-related questionnaires’ and also provides medical, dental, physiological and laboratory measurements.

The NHANES 2003–2004 and 2005–2006 cohorts’ data was examined in this study since there is detailed information regarding physical activity measured objectively by accelerometers, socioeconomic status, and chronic liver disease. The key exclusion criteria included: 1) were younger than 18 and older than 85; 2) had unspecified estimated wear time or had their data quality or device calibration flagged by NHANES; 3) consumed more than 7 drinks/week for females and more than 14 drinks/week for males; 4) had iron overload with transferrin saturation greater than 50%; 5) were pregnant; 6) had HIV; 7) had viral hepatitis C; 8) had hepatitis B; 9) had missing data for arthritis, type II diabetes, triglycerides, BMI, GGT or waist circumference; 10) were in the unspecified race category coded as Other.

Missing education values were classified into an additional category. Participants who reported to be of Mexican American and other Hispanic ethnicity were combined into a single Hispanic racial category. Participants with glucose plasma level greater than 126 mg were classified to have type II diabetes [26]. If fast glucose measurements were unavailable, type II diabetes was diagnosed by insulin level greater than 10 uU or the glycohemoglobin (HbA1c) levels greater than 6.5 uU [27]. Within the population of participants who were diagnosed with NAFLD, those with missing fibrosis index were further excluded from the fibrosis sub-analyses. Our exclusion criteria are consistent with the non-invasion NAFLD and advanced fibrosis diagnosis procedures based on the fatty liver index and FIB-4 index discussed in [28–32]. The fatty liver index and FIB-4 index were developed in the absence of all other causes like alcohol, drug-induced, autoimmune, viral, metabolic for chronic liver disease.

### Physical activity

The physical activity data in NHANES cohorts of 2003–2004 and 2005–2006 was collected via hip worn accelerometers (ActiGraph AM-7164). Participants were advised to wear the accelerometer for a period of 7 consecutive days except when sleeping or bathing. Non-wear time was defined as time intervals with at least 90 consecutive minutes of 0 activity counts and at most 2 minutes with counts between 0 and 100 [33]. Valid accelerometry wear days were identified using the R function exclude_accel in package rnhanesdata [34]. The total log activity count (TLAC) was defined as the sum of log(1+activity count) at the minute level. A light physical activity (LIPA) minute was defined as having activity counts equal to or more than 100 and less than 2020. A moderate to vigorous physical activity (MVPA) minute was defined as having an activity count greater or equal to 2020 [35, 36]. The total time spent in LIPA and MVPA and TLAC averaged across days with available accelerometry measures were used in analysis. Participants with either invalid or low-quality accelerometry data were excluded from the study.

### Occupation and socioeconomic status

Based on the reported annual family income (NHANES variable INDFMINC) the following categories were created: 1) below poverty, if the family income was less than $19,999; 2) above poverty, if the family income was more than $20,000; and (3) unknown income, if the participants refused to answer, had unknown or unreported income.

Participants’ occupational level of physical activity was defined by combining multiple questions from NHANES [37–39]. Participants were considered full-time employees if they responded as “working at a job or business” or “with a job or business but not at work” (NHANES question OCD150) in the week prior to the interview and the number of work hours per week was greater than 35, and part-time employees if the number of work hours per week was less than 35 (NHANES question OCQ210, OCQ180). Those who responded as “looking for work” or “not working at a job or business” (NHANES question OCD150) were classified as not employed. The remaining participants were categorized as not employed due to retirement or health reasons (NHANES question OCQ380). Using established classification system for 2003–2004 waves and extended classification system for 2005–2006 waves (see S1 File), reported occupations (NHANES question OCD240, OCD241) were categorized as professions with high, mixed and low physical activity [40, 41].

### Diagnosis of NAFLD and fibrosis

The diagnosis of NAFLD was established in the study cohort non-invasively using Fatty Liver Index (FLI), which has been validated extensively in patients with NAFLD [28–30]. Participants with the FLI > 60 were classified to have NAFLD.

The diagnosis of fibrosis was made within the population of NHANES participants with NAFLD using the fibrosis-4 index (FIB-4) [31]. Fibrosis was determined via FIB-4 with cutoff value > 1.37, suggesting presence of significant fibrosis [42].

### Statistical analysis

The NHANES 2003–2004 and 2005–2006 data files were downloaded and processed using the R package rnhanesdata [34, 35]. To ensure that results are generalizable to the entire US population each participant was assigned a survey weight, which corresponds to the number of individuals this participant represents in the population. Individuals’ survey weights were re-weighted based on the sub-population of the NHANES cohort selected for the purposes of this study via the reweight_accel() function in the rnhanesdata package [34].

#### Demographic summary

The complex-survey adjusted demographic and clinical characteristics of the participants were obtained using the tableone package in R.

#### NAFLD and fibrosis prediction models

To identify the best subset of variables that predicted NAFLD and FIB-4, a forward selection survey weighted multivariable logistic regression model was obtained by maximizing the 10-fold cross-validated AUC as optimization criteria. The models were fitted using the function svyglm() in the R package survey.

#### Candidate predictors selection

Variance inflation factor (VIF) was examined and predictors leading to VIF > 10 were excluded from analysis. Forward selection models were then re-run for final selection. For NAFLD the final selection was performed using the set of predictors age, gender, race, education, type II diabetes, CHF, stroke, arthritis, income, TLAC, MVPA, LIPA. For the fibrosis model the best predictors were selected from gender, race, education, BMI, type II diabetes, CHF, stroke, arthritis, income, TLAC, MVPA, LIPA.

#### Sociodemographic differences

Two-way ANOVA with interaction was performed to test for differences on MVPA, LIPA and TLAC by 1) race and occupational physical activity level; and 2) race and income level. Differences between individual racial, occupational physical activity and income level groups were assessed using Tukey’s Honest Significant Difference adjustment for multiple comparisons and the R function TukeyHSD. The independence between race and occupational physical activity, respectively income levels was tested using a chi-squared test of association for survey design. The upward trend in the amount of MVPA was tested using the Jonckheere-Terpstra rank-based test.

## Results

### Study cohort characteristics

**Fig 1:**
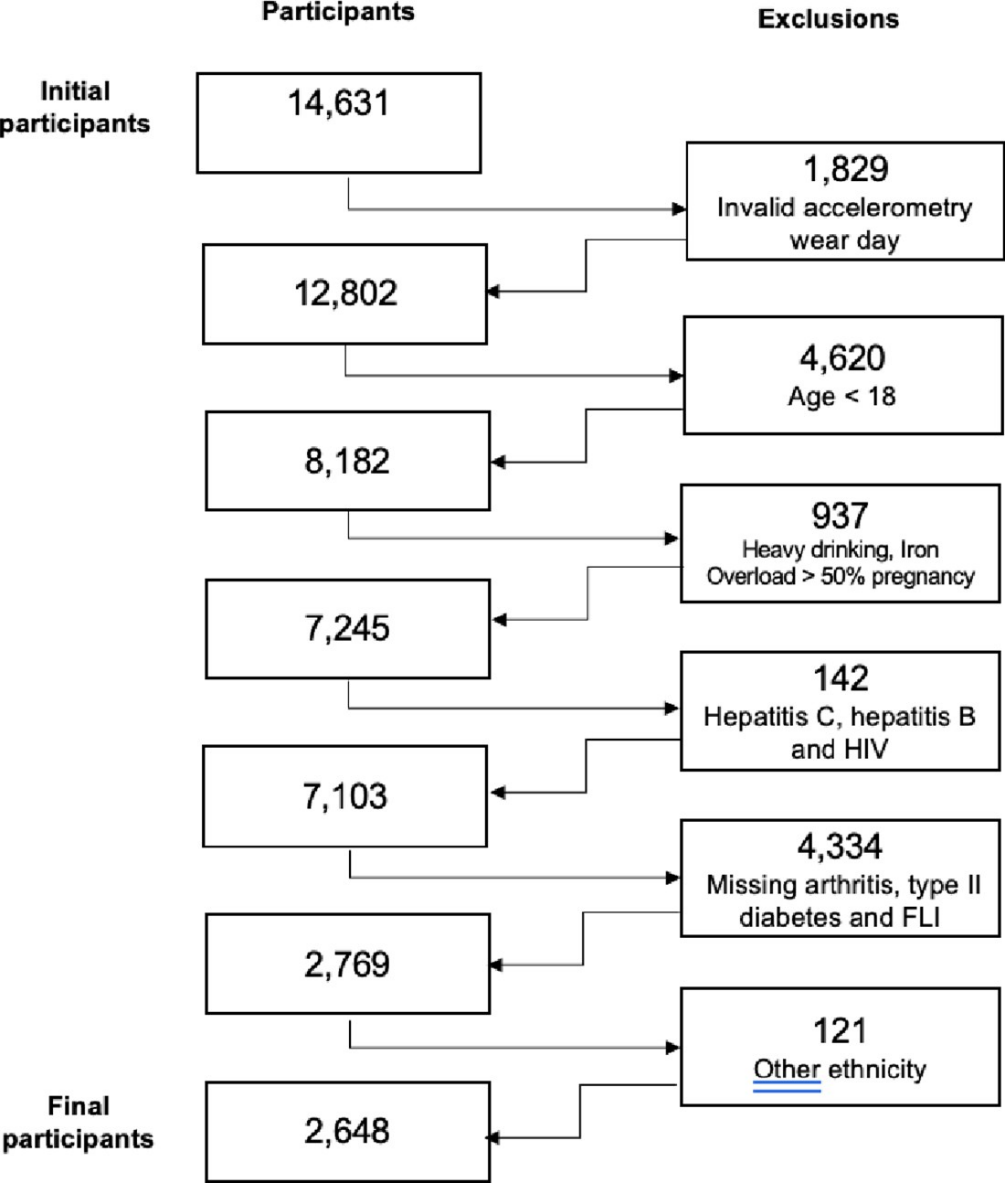
Flow chart showing the exclusion criteria.

A total of 14,631 NHANES in 2003–2004 and 2005–2006 participants had physical activity quantified via accelerometer. Of these, 12,802 participants had at least one valid accelerometry wear day (Fig 1). We excluded 4,620 participants who were younger than 18 years old, 481 heavy drinkers, 42 participants with iron overload greater than 50%, 414 pregnant women, 98 participants with hepatitis C, 17 participants with hepatitis B, 27 with HIV, 792 participants with missing arthritis information, 3,159 participants with missing type II diabetes information, 383 participants with missing the fatty liver index (FLI), and 121 participants with the ethnicity category Other than white, black or Hispanic. The final sample size was 2,648.

The clinical characteristics of the final study cohort are presented in Table 1. Compared to subjects without NAFLD, the subjects with NAFLD were older and more likely to be males. As expected, the distribution of metabolic comorbidities such as diabetes, obesity and hypertriglyceridemia were significantly higher as patients with NAFLD. When patients with NAFLD were stratified according to fibrosis status (Table 1), patients with significant fibrosis were more likely to be older, males, non-Hispanic whites with higher prevalence of metabolic co-morbid conditions. Regarding socioeconomic status (SES), no significant differences in poverty level were noted in patients with NAFLD [vs. non-NAFLD] or NAFLD with significant fibrosis [compared to those without significant fibrosis].

**Table 1:** Demographic and participant characteristics stratified by the NAFLD and significant fibrosis status. The total number of participants (N) is non-survey weighted, while the means (SD) or the totals (%) for each variable are survey-weighted.

		NAFLD			Significant fibrosis		
	Variable	Absent	Present	p-value	Absent	Present	p-value
		N = 1389	N = 1259		N = 923	N = 329	
**DEMOGRAPHIC**	Age	44.22 (16.79)	48.84(15.38)	<0.001	42.30(13.93)	67.93(10.88)	<0.001
	*Gender* female	872.2(59.4)	496.8(42.1)	<0.001	564.1(53.3)	83(42.8)	0.015
	*Race*			0.440			<0.001
	White	1135.4(77.3)	893.1(75.8)		784.5(74.2)	166.4(85.8)	
	Black	156.6(10.7)	145.8(12.4)		126.3(11.9)	15.8(8.1)	
	Hispanic	177.4(12.1)	139.8(11.9)		147.1(13.9)	11.8(6.1)	
**METABOLIC COMORBITIES**	Diabetes	47.6(3.2)	144.8(12.3)	<0.001	97.4(9.2)	38.9(20)	<0.001
	Stroke	22.5(1.5)	43.6(3.7)	0.002	23.8(2.2)	21.2(10.9)	<0.001
	*BMI*			<0.001			0.013
	Obese	100.7(6.9)	824.5(70)		792.4(74.9)	124.2(64)	
	Hypertension systolic	117.81(16.84)	125.36(16.47)	<0.001	122.68(14.73)	133.19(21.48)	<0.001
**SOCIOECONOMIC STATUS**	*Education*			0.053			0.153
	Less than high school	212.7(14.5)	203.7(17.3)		174.4(16.5)	46.4(23.9)	
	High school	351.4(23.9)	317.6(26.9)		285.1(26.9)	52(26.8)	
	More than high school	489.3(33.3)	386.5(32.8)		366.7(34.7)	53.6(27.6)	
	*Income*			0.197			0.066
	Above poverty	1174.9(80)	958.3(81.3)		856.6(81)	141.2(72.8)	
	Below poverty	259.6(17.7)	205.2(17.4)		188.5(17.8)	50.6(26.1)	
**LABORATORY**	AST	23.35(14.02)	26.56(14.16)	<0.001	24.73(8.79)	32.72(27.9)	<0.001
	ALT	21.62(10.87)	30.67(17.82)	<0.001	29.96(16.31)	30.31(22.26)	0.803
	Bilirubin total mg	0.78(0.29)	0.75(0.26)	0.014	0.7(0.25)	0.82(0.28)	<0.001
		(0 missing)	(1 missing)				
	Platelets	266.02(64.16)	276.38(73.18)	0.002	298.26(70.28)	211.47(51.05)	<0.001
	Total Cholesterol	193.25(37.72)	205.95(45.36)	<0.001	205.36(47.94)	200.14(42.33)	0.497
		(682 missing)	(617 missing)		(133 missing)	(477 missing)	
	HDL Cholesterol	59.59(15.81)	48.08(12.39)	<0.001	47.57(11.33)	53.21(15.85)	0.015
		(707 missing)	(642 missing)		(196 missing)	(446 missing)	
	LDL Cholesterol	113.34(33.82)	120.52(36.81)	<0.001	121.09(36.05)	113.95(38.95)	0.092
		(4 missing)	(73 missing)		(17 missing)	(56 missing)	
	Triglycerides (mg)	102.15(52.57)	195.22(144.13)	<0.001	191.63(143.2)	182.73(102.91)	0.455

### MVPA is the strongest predictor for NAFLD

The predictive model was developed to determine strength of relationship between NAFLD and physical activity. MVPA was the strongest predictor in the univariate logistic regression in the forward selection procedure with the highest AUC of 0.602. The model had CV-AUC of 0.687 and demonstrated that diagnosis of NAFLD was directly associated with Hispanic or black ethnicity, presence of diabetes and arthritis, and inversely with female gender, income level below poverty or unknown level, and MVPA (Table 2). There was a decrease of 43% in NAFLD risk with MVPA (OR = 0.569, p < 0.001). Females had a 62.4% lower risk for NAFLD (OR = 0.376, p < 0.001). Having diabetes or arthritis increased the risk for NAFLD (OR = 3.152, 1.774, p < 0.001). The NAFLD risk was not significantly associated with being black (OR = 1.24, p = 0.079) or Hispanic (OR = 1.212, p = 0.213) compared to white. While stroke was selected in forward regression models based on the CV-AUC criteria, it was not significantly associated with the risk of NAFLD.

**Table 2:** Estimated final model coefficient odds ratio (OR) for the NAFLD and significant fibrosis prediction models. For each model, the variables are arranged in the order they entered the forward selection model with the corresponding cumulative CV-AUC due to adding each variable into the model. For example, in the NAFLD model, the row labeled Gender indicates that gender is the second variable that was added to the model and the corresponding AUC includes MVPA and Gender.

NAFLD prediction model					Significant fibrosis prediction model				
Variable	OR Estimate	p-value	95% CI	Cumulative AUC	Variable	OR Estimate	p-value	95% CI	Cumulative AUC
MVPA	0.569	< 0.001	(0.470, 0.689)	0.602	MVPA	0.518	0.022	(0.296, 0.908)	0.705
Gender: female	0.376	< 0.001	(0.312, 0.454)	0.662	Arthritis: yes	2.863	<0.001	(1.922, 4.268)	0.729
Diabetes: yes	3.152	< 0.001	(2.197, 4.519)	0.677	Gender: female	0.450	0.001	(0.286, 0.707)	0.736
Arthritis: yes	1.774	< 0.001	(1.350, 2.330)	0.686	TLAC	0.296	0.017	(0.109, 0.803)	0.744
Race: black	1.247	0.079	(0.975, 1.595)	0.686	Diabetes: yes	1.606	0.075	(0.952, 2.711)	0.748
Race: Hispanic	1.212	0.213	(0.895, 1.641)		BMI: overweight	0.615	0.430	(0.184, 2.055)	0.752
Stroke: yes	1.548	0.117	(0.896, 2.675)	0.686	BMI: obese	0.343	0.124	(0.088, 1.342)	
Income: below poverty	0.867	0.168	(0.708, 1.062)	0.687	CHF: yes	1.470	0.112	(0.914, 2.360)	0.754
Income: unknown	0.434	0.053	(0.186, 1.009)		Income: below poverty	1.397	0.175	(0.862, 2.261)	0.755
					Income: unknown	0.792	0.802	(0.127, 4.920)	

### MVPA and socioeconomic status predicts significant fibrosis

The advanced fibrosis prediction model had a CV-AUC of 0.755 and included MVPA, arthritis, gender, TLAC, type II diabetes, BMI, CHF and income status (Table 2). Presence of significant fibrosis was inversely associated with MVPA, female gender and TLAC and positively associated with arthritis. The prediction model for significant fibrosis showed that there was a 48% and a 70% decrease in significant fibrosis risk with MVPA (OR = 0.518, p = 0.022) and TLAC (OR = 0.296, p = 0.017), respectively. Females had a 55% lower risk for significant fibrosis (OR = 0.45, p = 0.001). There was a high risk for significant fibrosis in subjects with arthritis (OR = 2.863, p < 0.001). While diabetes or CHF were selected in a forward regression model based on the CV-AUC criteria, the risk for significant fibrosis was not statistically associated with these variables (OR = 1.606,1.470, p = 0.075, 0.112). The risk for significant fibrosis was not statistically associated with a reduced risk of overweight or obesity (OR = 0.615, 0.343, p = 0.430, 0.124), and with an increased below poverty income (OR = 1.397, p = 0.175).

### Individuals with NAFLD and significant fibrosis engage in less physical activity

**Fig 2:**
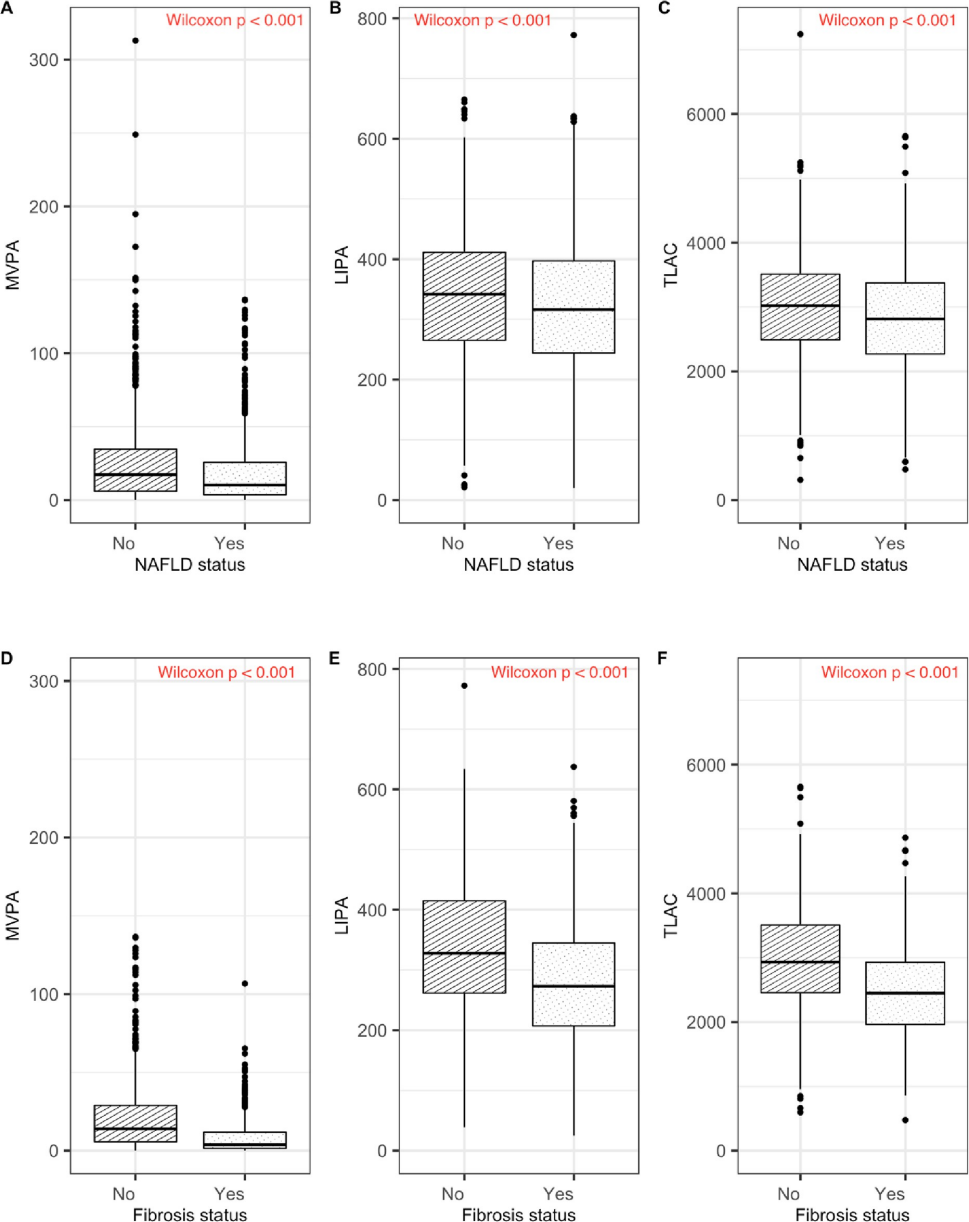
MVPA, LIPA and TLAC physical activity summaries by NAFLD (top panel) and significant fibrosis (bottom panel) status. In panels A-F the Wilcoxon test showed a p-value < 0.001 indicating differences in physical activity between those who have NAFLD or fibrosis and those who do not have the disease.

Based on objectively measured accelerometry data, participants with NAFLD spent less time in MVPA, LIPA and had lower TLAC (Fig 2A–2C). Similarly, among those who had NAFLD, the patient with significant fibrosis had significantly lower MVPA, LIPA and had lower TLAC (Fig 2D and 2E).

### Variable physical activity based on ethnicity

Race and occupational physical activity are associated variables for participants with NAFLD (p < 0.001). Employed white and black individuals with NAFLD had predominantly occupations that involved low physical activity (high PA: 11% white and 11.6% black; mixed PA: 23.1% white and 23.1% black; low PA: 30.9% white and 28.5% black). Employed Hispanics with NAFLD had occupations that were distributed similarly among the three types of PA intensity categories (high PA: 24.9%, mixed PA: 25.1%, low PA: 24.0%).

Among the participants with NAFLD, race, occupational PA level and their interaction were significantly associated with different levels of MVPA (p < 0.001 for race and occupational PA, p = 0.021 for their interaction in the 2-way ANOVA test). The racial significant differences in MVPA were between Hispanic and white (p < 0.001), Hispanic and black (p = 0.008) with Hispanics having increased levels of MVPA than black and white groups.

**Fig 3:**
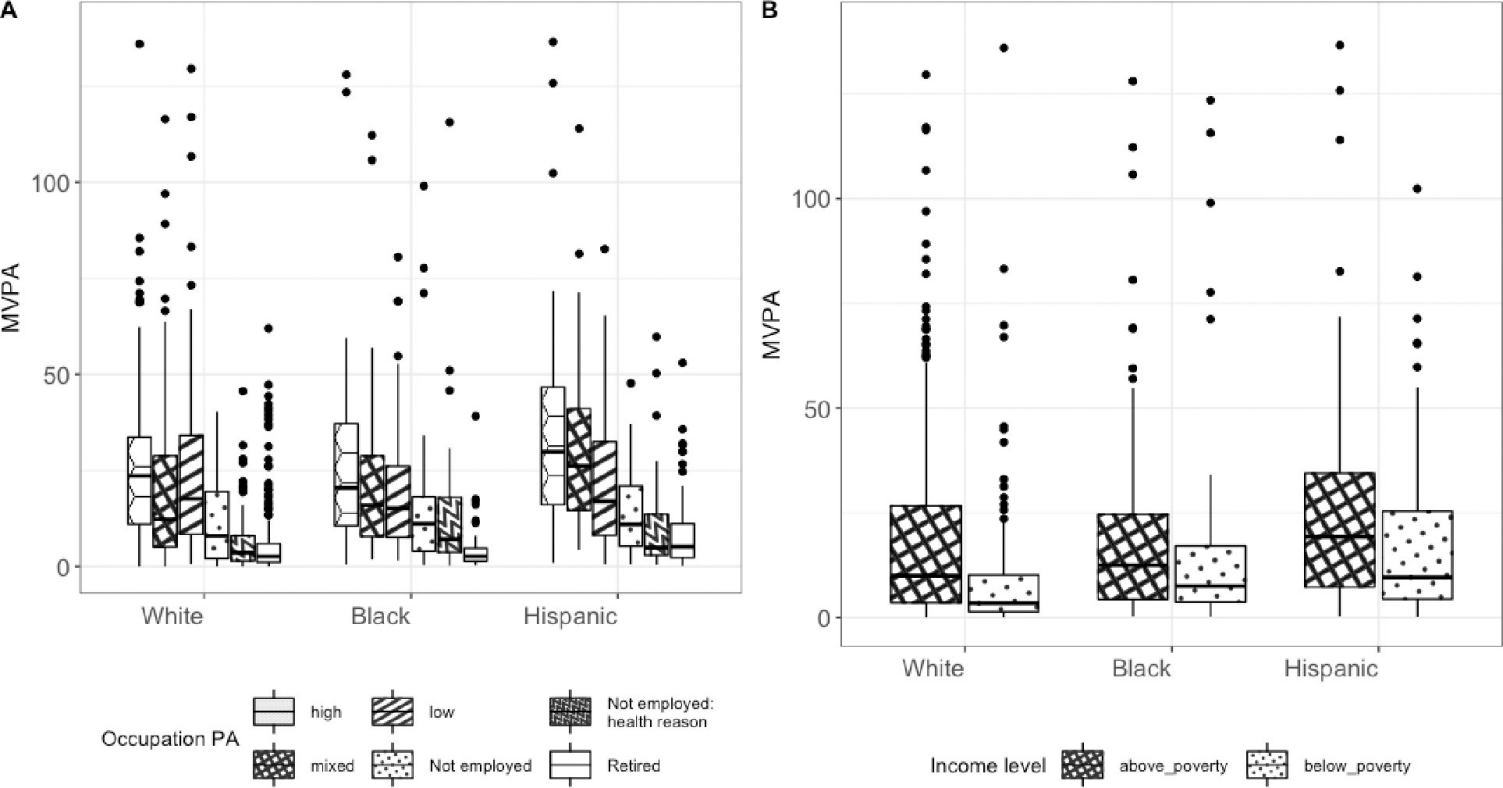
Boxplots of MVPA by occupational PA levels and of income levels for white, black and Hispanic participants with NAFLD.

Individual statistically significant pair-wise differences across races and occupational status are shown in Fig 3 (panel A) and in S2 Table of S1 File. MVPA levels were higher for Hispanics with jobs characterized as having a low PA level when compared to other racial groups. For Hispanics with jobs characterized as having a mixed level of PA, their MVPA levels were higher when compared to white but not black participants. MVPA levels were higher for Hispanics with jobs characterized as having high levels of PA when compared to those of any race and unemployed due to any reason.

### The interaction between physical activity, NAFLD and socioeconomic status

Race and income levels were associated variables for participants with NAFLD (p < 0.001). A higher percentage of the white, black and Hispanic individuals with NAFLD are above the poverty line (15% below poverty vs 84% above poverty for whites; 25% below poverty vs 74% above poverty for blacks; 27% below poverty vs 71% above poverty for Hispanics).

Next, we evaluated the impact of poverty on MVPA and noted that MVPA was significantly higher in patients who were living above the poverty level when compared to those below poverty level across all ethnicities (Fig 3, **panel B**). A stepwise increase in MVPA was noted in patients with NAFLD living below the poverty threshold from whites to blacks to Hispanics (p < 0.001 from Jonckheere-Terpstra rank-based test).

Among participants with NAFLD, race and poverty were significantly associated with different levels of MVPA but their interaction was not significant (p < 0.001 for race and income, p = 0.157 for their interaction in 2-way ANOVA). The racial significant differences in MVPA were between Hispanic and white (p < 0.001), Hispanic and black (p = 0.028) with Hispanics having increased levels of MVPA compared to black and white groups.

### Higher proportion of Hispanic patients with fibrosis are employed and have increased levels of MVPA

Race and occupational physical activity are associated variables for participants with significant fibrosis (p < 0.001). White and black individuals with fibrosis were predominantly retired (51.3% white and 39.3% black). Those who were employed had occupations that involved low physical activity (high PA: 2.6% white and 5.2% black; mixed PA: 13.4% white and 9.9% black; low PA: 16.1% white and 19.2% black). Black participants with significant fibrosis were 20.3% unemployed due to health problems (9.3% white and 11.3% Hispanic). For Hispanic participants with significant fibrosis 28% were not employed, followed by 24.9% retired. They also had occupations that involved 17.5% high PA, 9.5% mixed PA and 8.8% low PA.

Among participants with significant fibrosis, race and occupational PA were significantly associated with different levels of MVPA but their interaction was not significant (p = 0.004 for race, p < 0.001 for occupational PA, p = 0.179 for their interaction in 2-way ANOVA). The racial significant differences in MVPA were between Hispanic and white (p = 0.017), Hispanic and black (p = 0.005) with Hispanics having increased levels of MVPA than black and white groups.

**Fig 4:**
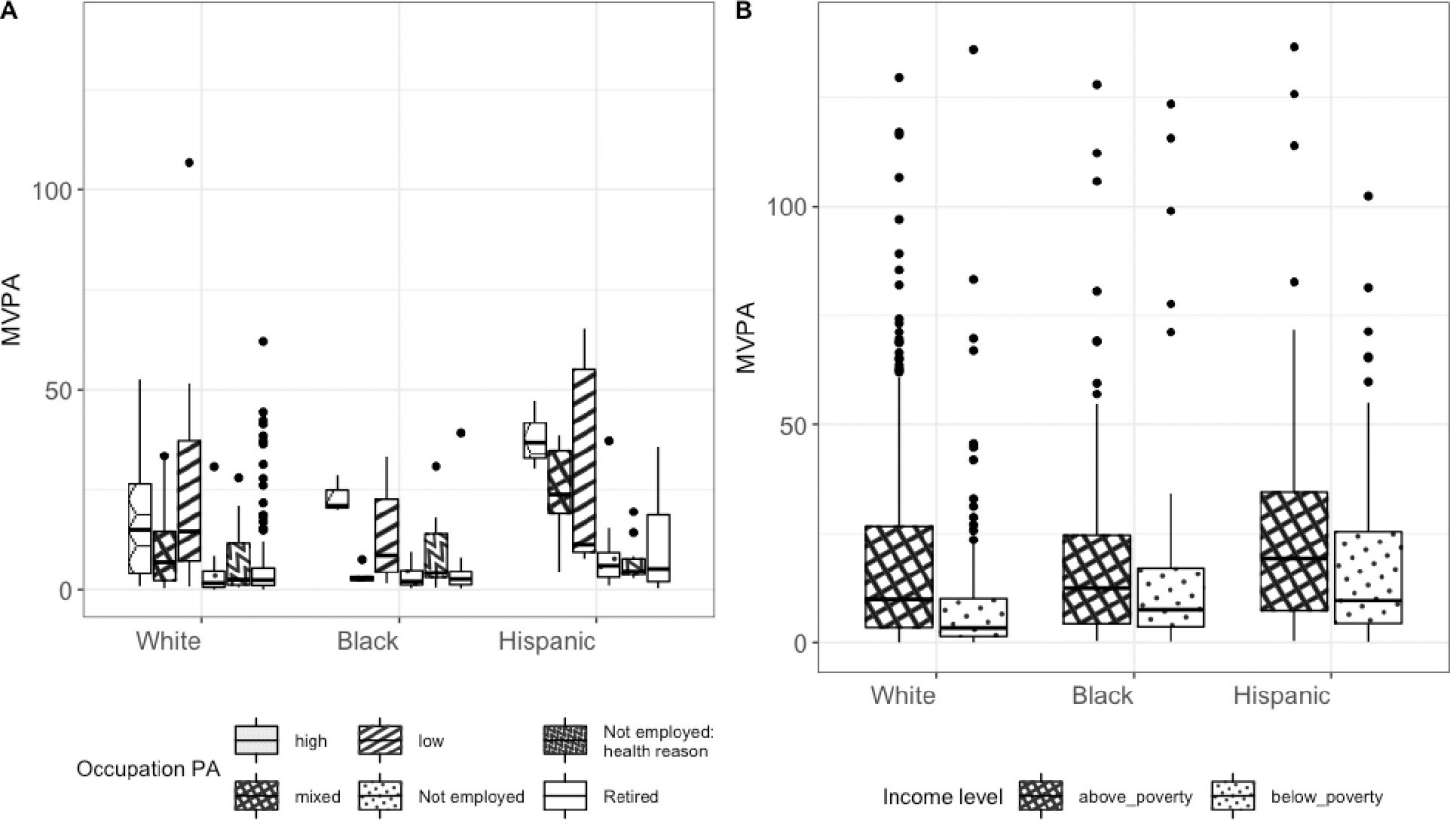
Boxplots of MVPA by occupational PA levels and of income levels for white, black and Hispanic participants with significant fibrosis.

Individual statistically significant pair-wise differences across races and occupational status are shown in Fig 4 (**panel A**) and in S2 Table of S1 File.

### MVPA is related to increased likelihood of advanced fibrosis in patients living above poverty

Race and income are not associated variables for participants with significant fibrosis (p = 0.711). A higher percentage of the white, black and Hispanic individuals with fibrosis were above the poverty line (25.2% below poverty vs 73.9% above poverty for whites; 30.4% below poverty vs 67.3% above poverty for blacks; 33.3% below poverty vs 65.2% above poverty for Hispanics).

Next, we evaluated the impact of poverty on MVPA and noted that MVPA was significantly higher in patients who were living above the poverty level when compared to those below poverty level across all ethnicities (Fig 4, panel B). A stepwise increase in MVPA was noted in patients with fibrosis living below the poverty threshold from whites to blacks to Hispanics (p = 0.001 from Jonckheere-Terpstra rank-based test).

Among participants with fibrosis, race and income were significantly associated with different levels of MVPA but their interaction was not significant (p = 0.016 for race, p = 0.004 for income, p = 0.384 for their interaction in 2-way ANOVA). The racial significant differences in MVPA were between Hispanic and white (p-value = 0.039), Hispanic and black (p = 0.021) with Hispanics having increased levels of MVPA than black and white groups.

## Discussion

The current study provides insights into the relationship between physical activity and the socioeconomic level in patients with NAFLD and significant fibrosis. Using physical activity as measured by accelerometers in the large population National Health and Examination Survey (NHANES) 2003–2006 cohort, we developed predictive models for NAFLD and significant fibrosis. Our findings show that individuals with NAFLD and further NAFLD with significant fibrosis engage in reduced physical activity. The levels of physical activity are further impacted by race and type of occupation.

We demonstrate that MVPA, race, presence of diabetes and arthritis, gender and income level illustrate that higher levels of intense physical activity significantly lower the risk for NAFLD. Furthermore, our study shows that among individuals with NAFLD, the risk for significant fibrosis decreases with more time spent in MVPA, TLAC. These data corroborate previous findings based on self-reports that physical activity was inversely associated with NAFLD and associated significant fibrosis [8], and higher intensities of physical activity showed larger protective effects [43–45]. Additionally, our predictive models quantify the relationship between the time engaged in MVPA, thereby, providing foundational data to better investigate these relationships in larger datasets that included both objective and self-reported measures of physical activity. These results further suggest the potential for the use of accelerometers as disease-monitoring biomarkers and support their future development for these indications.

We also specify that the amount of physical activity differs by race and socioeconomic status. NAFLD is a complex metabolic disease that is further influenced by a multitude of socio-environmental factors like individual, social (race, education, income), environmental (restricted access to healthy food), macro-level (media, government policy) [46]. In our study, NAFLD and advanced fibrosis were presented in higher proportions of Hispanic patients, confirming the role of other environmental and genetic factors. Furthermore, among participants with NAFLD, white and black individuals with significant fibrosis were predominantly retired or were employed in occupations that involved lower levels of physical activity. Interestingly, race and income were associated variables for participants with NAFLD but not with presence of significant fibrosis. These results confirm previous evidence that genetic factors play an important role in NAFLD and significant fibrosis [47]. We further summarize that while higher physical activity levels are protective for NAFLD and fibrosis, the time spent in physical activity should be larger in Hispanic individuals to reduce the risk for NAFLD. These findings have important implications suggesting that NAFLD prevention and management guidelines should be developed considering differences between racial groups.

The study results must be interpreted within the context of study limitations. First, we used non-invasive FLI and FIB-4 measures to define patients with NAFLD and significant fibrosis. Therefore, some individuals may be incorrectly classified. However, it is not feasible to perform liver biopsy in a population based study given the cost and invasive nature of a liver biopsy. Moreover, this study also reflects a ‘real world’ cohort as opposed to previously published cohorts that are limited by ascertainment bias, thereby, allowing for broader implications of the study findings. Another important limitation is the use of 2003–2006 NHANES data. While the new waves of NHANES data are available that include better diagnostic assessment of liver (i.e. vibration controlled transient elastography), we selected the older data because of availability of accelerometry data measured by Actigraph step counts verified cutoffs for sedentary, LIPA and MVPA times. In our prediction models, arthritis is a statistically significant variable and this is likely because these are older patients, and the rates of arthritis, particularly osteoarthritis, increases with age, BMI and likely limits physical activity.

Our analysis showed that physical activity and socioeconomic levels are associated with NAFLD and advanced fibrosis differently for Hispanic, black and white participants. The goal of this study was to increase awareness of health disparities in different populations affected by liver diseases like NAFLD and clinically significant fibrosis. Potential recommendations for improving the situation include changes at the individual, institutional, community and societal level. Disadvantaged groups may benefit from an increased accessibility to health care, educational programs on nutrition and lifestyle, management of physical activity, recreational facilities.

In summary, the major strengths of our study is the use of objectively-measured physical activity in the large nationally-representative population. The current study demonstrates racial and occupational disparities among the patients with NAFLD and significant fibrosis, which implicate the need for race-specific guidelines for NAFLD prevention and management. Since accelerometers allow to measure physical activity effortlessly and objectively in a natural living environment, wearable technology can be used to design targeted interventions to monitor diet, physical activity and other health comorbidities in vulnerable groups affected by liver diseases. NHANES is a rich data resource on the diverse population of US, and in UK similar information is collected in the UK Biobank data set. Research questions regarding the consistency of our results compared with UK Biobank could be examined in the future studies.

## Supporting information

S1 File (DOCX)
