# Supplementary material for: Socioeconomic status and health disparities drive differences in accelerometer-derived physical activity in fatty liver disease and significant fibrosis
Source: PLoS One. 2024 May 9;19(5):e0301774. doi: 10.1371/journal.pone.0301774 (PMC11081311; doi:10.1371/journal.pone.0301774)
Supplement: S1 File — (DOCX) [file pone.0301774.s001.docx]

**SOCIOECONOMIC STATUS AND HEALTH DISPARITIES DRIVE DIFFERENCES IN ACCELEROMETER-DERIVED PHYSICAL ACTIVITY IN FATTY LIVER DISEASE AND SIGNIFICANT FIBROSIS**

Lucia Tabacu, Sajag Swami, Mark Ledbetter, Mohammad Shadab Siddiqui and Ekaterina Smirnova

**Supplemental materials**

We calculated new variables as type II diabetes, the fatty liver index (FLI), the fibrosis-4 index (FIB-4), occupational physical activity, and income level. A participant was considered to have type II diabetes when the glucose plasma level was greater than 126 mg (28). For those subjects without fast glucose measurements available, type II diabetes was diagnosed if they had insulin level greater than 10 uU or the glycohemoglobin (HbA1c) levels were greater than 6.5 uU. The fatty liver index (FLI) was calculated based on levels of triglycerides (mg), BMI (kg/$m^{2}$), Gamma glutamyl transferase (GGT, U/L), waist circumference (cm) according to the formula

$FLI= \frac{\eta}{(1+\eta)}\times100$, where $\eta=exp(0.953*log\left( triglycerides\left( mg \right) \right)+0.139*BMI+0.718*log\left( GGT \right)+0.053*log\left( waist circumference \right)-15.745)$ and the nonalcoholic fatty liver disease (NAFLD) was detected if the FLI was greater than 60 (37, 39).

The fibrosis-4 index (FIB-4) was obtained only for those participants who were diagnosed with NAFLD and it was calculated as FIB-4 = $\frac{Age (years)*AST (U/L)}{platelets ({10}^{9}/L)*\sqrt{ALT(U/l)}}$.

**Table S1:** Classification of occupational categories for NHANES 2003-2004, 2005-2006

| ***NHANES 2003-2004*** |  | ***NHANES 2005-2006*** |  |
| --- | --- | --- | --- |
| **Occupational categories classified as having high PA (n = 7)** | **Code corresponding to each category** | **Occupational categories classified as having high PA (n = 7)** | **Code corresponding to each category** |
| Waiters and waitresses | 19 | Personal Care, Service Occupations | 15 |
| Cleaning and building service | 23 | Installation, Maintenance, Repair Occupations | 20 |
| Farm and nursery workers | 26 | Farming, Fishing, Forestry Occupations | 18 |
| Construction trades | 30 | Building & Grounds Cleaning, Maintenance Occupations | 14 |
| Construction laborers | 37 | Construction, Extraction Occupations | 19 |
| Laborers, except construction | 38 | Production Occupations | 21 |
| Freight, stock, and material movers (hand) | 39 | Armed Forces | 23 |
|  |  | Food Preparation, Serving Occupations | 13 |
| **Occupational categories classified as having low PA (n = 10)** |  |  |  |
| Executive, administrators, and managers | 1 | Management | 1 |
| Management related | 2 | Business, Financial Operations | 2 |
| Engineers, architects and scientists | 3 | Computer, Mathematical Occupations | 3 |
| Teachers | 5 | Architecture, Engineering Occupations | 4 |
| Secretaries, stenographers, and typists | 12 | Education, Training, Library Occupations | 8 |
| Information clerks | 13 | Legal Occupations | 7 |
| Records processing | 14 | Life, Physical, Social Science Occupations | 5 |
| Material recording, scheduling, and distributing clerks | 15 |  |  |
| Miscellaneous administrative support | 16 | Office, Administrative Support Occupations | 17 |
| Motor vehicle operators | 35 | Transportation, Material Moving Occupations | 22 |
| **Occupational categories classified as having unclassifiable (mixed) PA (n = 23)** |  |  |  |
| Health diagnosing, assessing and treating | 4 | Healthcare Practitioner, Technical Occupations | 10 |
|  |  | Healthcare Support Occupations | 11 |
| Writers, artists, entertainers, and athletes | 6 | Arts, Design, Entertainment, Sports, Media Occupations | 9 |
| Other professional specialty | 7 | Community, Social Services Occupations | 6 |
| Technicians and related support | 8 | Protective Service Occupations | 12 |
| Supervisors and proprietors, sales | 9 |  |  |
| Sales representatives, finance, business, & commodities ex. Retail | 10 | Sales & Related Occupations | 16 |
| Sales workers, retail and personal services | 11 |  |  |
| Private household | 17 |  |  |
| Protective service | 18 |  |  |
| Cooks | 20 |  |  |
| Miscellaneous food preparation and service | 21 |  |  |
| Health service | 22 |  |  |
| Personal service | 24 |  |  |
| Farm operators, managers, and supervisors | 25 |  |  |
| Related agricultural, forestry, and fishing | 27 |  |  |
| Vehicle and mobile equipment mechanics and repairers | 28 |  |  |
| Other mechanics and repairers | 29 |  |  |
| Extractive and precision production | 31 |  |  |
| Textile, apparel, and furnishings machine operators | 32 |  |  |
| Machine operators, assorted materials | 33 |  |  |
| Fabricators, assemblers, inspectors, and samplers | 34 |  |  |
| Other transportation and material moving | 36 |  |  |
| Other helpers, equipment cleaners, hand packagers and laborers | 40 |  |  |
| Military | 41 |  |  |

**Table S2:** Significant pair-wise comparisons for race, occupational PA (high, mixed, low, not employed, not employed health reason, retired levels) and their interaction for NAFLD participants

| Variables | Difference | p-value adj |
| --- | --- | --- |
| Mixed occupational PA– high occupational PA | -7.069 | 0.002 |
| Low occupational PA - high occupational PA | -7.101 | 0.001 |
| Not employed – high occupational PA | -16.722 | < 0.001 |
| Retired – high occupational PA | -22.888 | < 0.001 |
| Not employed health reason – high occupational PA | -20.255 | < 0.001 |
| Not employed – mixed occupational PA | -9.653 | < 0.001 |
| Retired – mixed occupational PA | -15.818 | < 0.001 |
| Not employed health reasons – mixed occupational PA | -13.185 | < 0.001 |
| Not employed – low occupational PA | -9.621 | < 0.001 |
| Retired – low occupational PA | -15.786 | < 0.001 |
| Not employed health reasons – low occupational PA | -13.153 | < 0.001 |
| Retired – not employed | -6.165 | 0.016 |
| White: not employed – white: high occupational PA | -18.000 | < 0.001 |
| Hispanic: not employed – white: high occupational PA | -14.687 | 0.008 |
| White: retired – white: high occupational PA | -22.494 | < 0.001 |
| Black: retired – white: high occupational PA | -24.543 | < 0.001 |
| Hispanic: retired – white: high occupational PA | -19.267 | < 0.001 |
| White: not employed health reason – white: high occupational PA | -22.146 | < 0.001 |
| Black: not employed health reason – white: high occupational PA | -14.560 | 0.019 |
| Hispanic: not employed health reason – white: high occupational PA | -18.207 | < 0.001 |
| White: not employed – black: high occupational PA | -17.095 | 0.003 |
| White: retired – black: high occupational PA | -21.589 | < 0.001 |
| Black: retired – black: high occupational PA | -23.639 | < 0.001 |
| Hispanic: retired – black: high occupational PA | -18.362 | < 0.001 |
| White: not employed health reason – black: high occupational PA | -21.241 | < 0.001 |
| Hispanic: not employed health reason – black: high occupational PA | -17.302 | 0.011 |
| White: mixed occupational PA – Hispanic: high occupational PA | -14.715 | < 0.001 |
| White: low occupational PA – Hispanic: high occupational PA | -9.813 | 0.031 |
| Black: low occupational PA – Hispanic: high occupational PA | -14.460 | < 0.001 |
| Hispanic: low occupational PA – Hispanic: high occupational PA | -11.597 | 0.036 |
| White: not employed – Hispanic: high occupational PA | -23.143 | < 0.001 |
| Black: not employed – Hispanic: high occupational PA | -15.614 | 0.016 |
| Hispanic: not employed – Hispanic: high occupational PA | -19.830 | < 0.001 |
| White: retired – Hispanic: high occupational PA | -27.637 | < 0.001 |
| Black: retired – Hispanic: high occupational PA | -29.686 | < 0.001 |
| Hispanic: retired – Hispanic: high occupational PA | -24.410 | < 0.001 |
| White: not employed health reason – Hispanic: high occupational PA | -27.289 | < 0.001 |
| Black: not employed health reason – Hispanic: high occupational PA | -19.703 | < 0.001 |
| Hispanic: not employed health reason – Hispanic: high occupational PA | -23.350 | < 0.001 |
| Hispanic: mixed occupational PA – white: mixed occupational PA | 10.663 | 0.018 |
| White: retired – white: mixed occupational PA | -12.921 | < 0.001 |
| Black: retired – white: mixed occupational PA | -14.971 | < 0.001 |
| White: not employed health reason – white: mixed occupational PA | -12.573 | 0.001 |
| White: retired – black: mixed occupational PA | -16.330 | < 0.001 |
| Black: retired – black: mixed occupational PA | -18.380 | < 0.001 |
| Hispanic: retired – black: mixed occupational PA | -13.103 | 0.020 |
| White: not employed health reason – black: mixed occupational PA | -15.982 | < 0.001 |
| White: not employed – Hispanic: mixed occupational PA | -19.091 | < 0.001 |
| Hispanic: not employed – Hispanic: mixed occupational PA | -15.778 | 0.001 |
| White: retired – Hispanic: mixed occupational PA | -23.585 | < 0.001 |
| Black: retired – Hispanic: mixed occupational PA | -25.635 | < 0.001 |
| Hispanic: retired – Hispanic: mixed occupational PA | -20.358 | < 0.001 |
| White: not employed health reason – Hispanic: mixed occupational PA | -23.237 | < 0.001 |
| Black: not employed health reason – Hispanic: mixed occupational PA | -15.651 | 0.004 |
| Hispanic: not employed health reason – Hispanic: mixed occupational PA | -19.299 | < 0.001 |
| White: not employed – white: low occupational PA | -13.329 | < 0.001 |
| White: retired – white: low occupational PA | -17.823 | < 0.001 |
| Black: retired – white: low occupational PA | -19.873 | < 0.001 |
| Hispanic: retired – white: low occupational PA | -14.596 | < 0.001 |
| White: not employed health reason – white: low occupational PA | -17.475 | < 0.001 |
| Hispanic: not employed health reason – white: low occupational PA | -13.537 | 0.006 |
| White: retired – black: low occupational PA | -13.176 | < 0.001 |
| Black: retired – black: low occupational PA | -15.226 | < 0.001 |
| White: not employed health reason – black: low occupational PA | -12.828 | 0.004 |
| White: retired – Hispanic: low occupational PA | -16.039 | < 0.001 |
| Black: retired – Hispanic: low occupational PA | -18.088 | < 0.001 |
| Hispanic: retired – Hispanic: low occupational PA | -12.812 | 0.016 |
| White: not employed health reason – Hispanic: low occupational PA | -15.691 | < 0.001 |


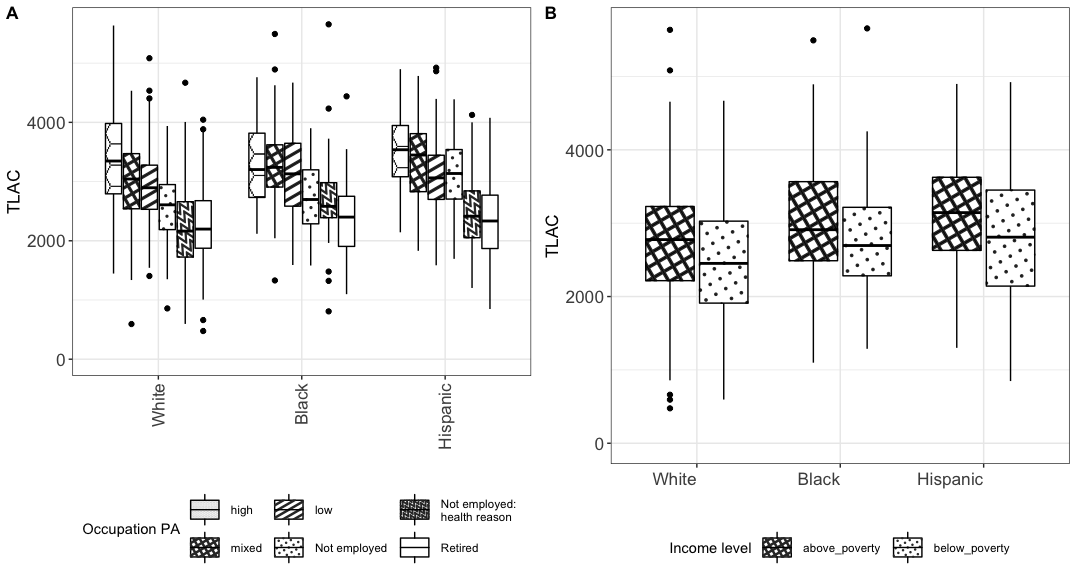


**Figure S1:** Boxplots of TLAC by occupational PA levels and of income levels for white, black and Hispanic participants with NAFLD


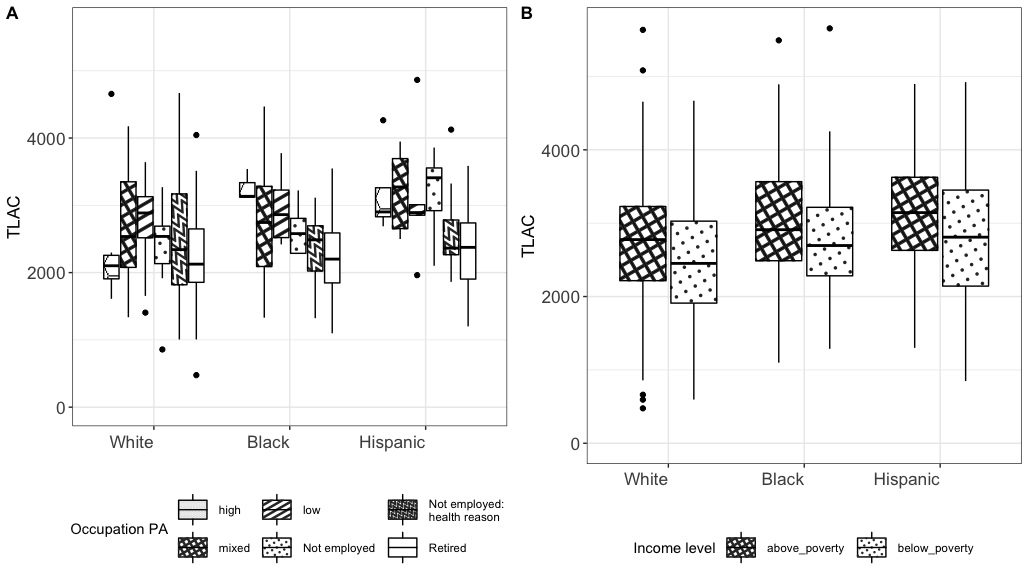


**Figure S2:** Boxplots of TLAC by occupational PA levels and of income levels for white, black, and Hispanic participants with fibrosis


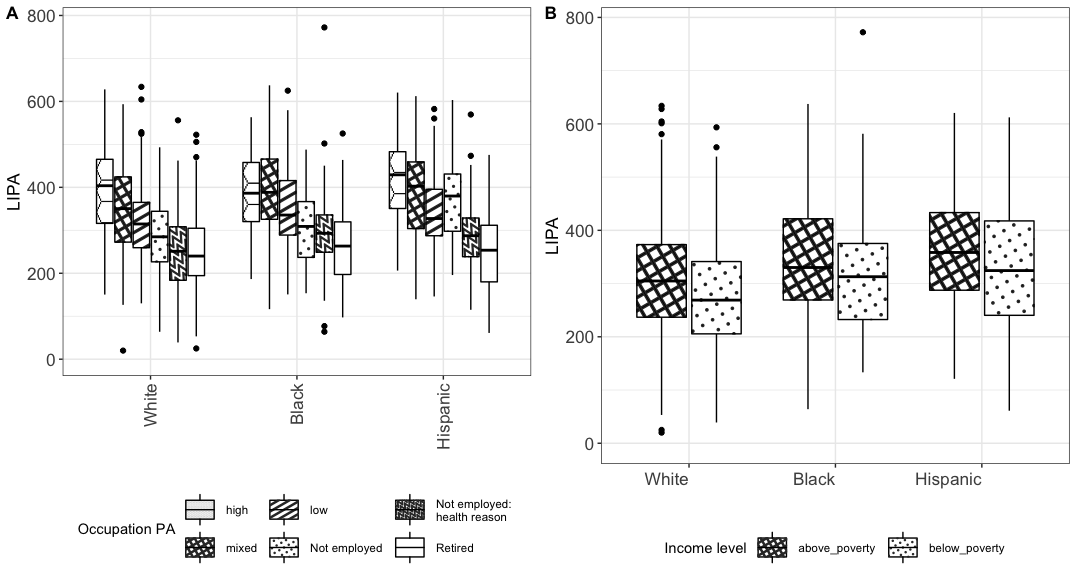


**Figure S3:** Boxplots of LIPA by occupational PA levels and of income levels for white, black and Hispanic participants with NAFLD


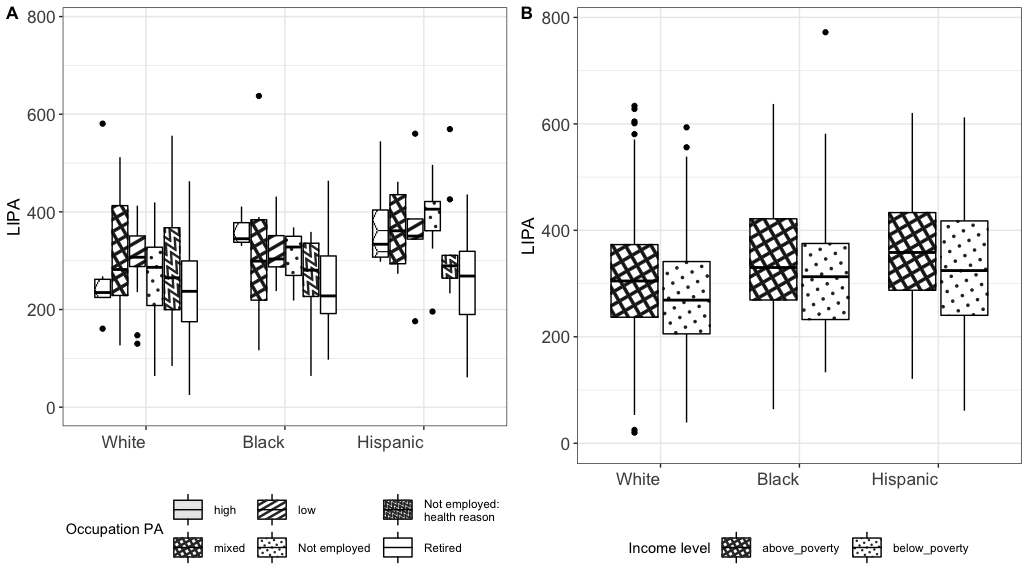


**Figure S4:** Boxplots of LIPA by occupational PA levels and of income levels for white, black and Hispanic participants with fibrosis
